# Beyond angiogenesis: integrating ferroptosis and metabolic rewiring for next-generation RCC therapy

**DOI:** 10.3389/fonc.2026.1800566

**Published:** 2026-07-15

**Authors:** Xu Chen, Yaxi Peng, Ye Tian

**Affiliations:** Department of Urology, Chongqing Wulong People’s Hospital, Chongqing, Wulong, China

**Keywords:** cancer metabolism, chromophobe renal cell carcinoma, ferroptosis, glutathione metabolism, iron homeostasis, lipid peroxidation, mitochondrial dysfunction, renal cell carcinoma

## Abstract

The diverse group of cancers known as renal cell carcinoma can be identified by their differing genetic mutations and distinct patterns of metabolism. Recent evidence suggests that changes in cellular metabolism have a major impact on renal cancer cell viability, therapeutic resistance and disease progression. Chromophobe renal cell carcinoma (chRCC) appears to be one of the subtypes of renal cell carcinoma that exhibits a most unique pattern of metabolism; chRCC cells display both mitochondrial abnormalities and an altered redox state, in addition to an abnormality in the regulation of iron. These abnormalities suggest that there are metabolic vulnerabilities associated with the development of renal tumors that are different from those seen in clear cell RCC and papillary RCC. A recently described process, called ferroptosis, is a highly regulated process of cell death mediated by iron-dependent lipid peroxidation. Ferroptosis appears to be particularly relevant in the context of renal cancer because it is metabolically regulated and requires intact glutathione metabolism, mitochondrial function, lipid composition, and iron homeostasis. All of these processes are disrupted in renal tumors. In this review, we will synthesize our present understanding of the changes in metabolism that occur in renal cell carcinoma, with a focus on chromophobe renal cell carcinoma, and critically examine the mechanisms underlying and the therapeutic potential of inducing ferroptosis. Through integration of findings from experimental models, translational studies and emerging clinical observations, we will investigate how disruption of mitochondrial function, glutathione dependence, and regulation of iron homeostasis contribute to sensitization of renal cancer cells to ferroptotic death. Finally, we will provide a discussion of pharmacological approaches to target these pathways, mechanisms of resistance to such approaches, and issues related to the heterogeneity of renal tumors and clinical translation. Ultimately, this review will provide a broad overview of ferroptosis as a metabolically driven therapeutic strategy for renal cell carcinoma and highlight areas for future investigation and clinical development.

## 1 Introduction

Renal cell carcinoma (RCC) comprises a heterogeneous group of malignancies with a rising global incidence and a substantial clinical burden (1–3). Over the past decade, advances in angiogenesis inhibitors and immune checkpoint blockade have reshaped the therapeutic landscape for clear cell RCC (ccRCC). However, inconsistent response rates and the emergence of acquired resistance remain major clinical challenges (4–6). More importantly, these systemic therapies have shown limited efficacy in non-clear cell variants, including chromophobe RCC (chRCC). Although localized chRCC is often associated with a relatively favorable prognosis, metastatic chRCC can behave aggressively and still lacks tailored, evidence-based targeted therapies (7–11). This disparity underscores the need to identify novel therapeutic targets rooted in the intrinsic biological vulnerabilities of distinct RCC subtypes.

Accumulating evidence suggests that metabolic reprogramming is a defining feature of RCC (12–14). Arising from highly active renal tubular epithelial cells, these tumors must continually adapt to oncogenic stress and fluctuating microenvironmental conditions. To do so, they extensively reprogram central carbon, lipid, and iron metabolism (14–18). Importantly, this metabolic rewiring is not uniform across all RCCs, but instead displays pronounced subtype specificity. Whereas ccRCC is largely characterized by VHL/HIF-associated pseudo-hypoxic reprogramming, chRCC exhibits a distinct biology linked to its origin from intercalated cells and to widespread chromosomal losses (19–22). Increasing evidence suggests that these genetic and cellular features may place chRCC cells under substantial redox stress. In this context, metabolic adaptation likely serves not only to sustain proliferation, but also to buffer oxidative pressure, thereby creating potentially targetable biochemical bottlenecks (23–25).

Ferroptosis, a regulated form of necrotic cell death driven by iron-dependent lipid peroxidation, has recently emerged as a compelling avenue for exploiting such metabolic vulnerabilities (26). Unlike apoptosis, ferroptosis is tightly governed by cellular metabolic state, particularly the balance among polyunsaturated fatty acid availability, redox buffering capacity, and iron homeostasis (25, 26). This close link to redox biology makes ferroptosis an especially relevant pathway to consider in chRCC. Histologically, chRCC is characterized by marked accumulation of dysfunctional mitochondria and defects in respiratory chain function (8, 10, 11). Because redox homeostasis in this subtype may be under sustained pressure, chRCC cells may rely heavily on glutathione-dependent antioxidant defenses. These features raise the possibility that chRCC could represent a particularly informative context in which to investigate ferroptosis-related vulnerabilities.

Despite this rationale, current discussions of ferroptosis in kidney cancer have largely focused on RCC as a general category or on pseudo-hypoxia in ccRCC. By contrast, the possible relevance of ferroptosis to chRCC, particularly in relation to its profound mitochondrial abnormalities and redox stress, has received comparatively limited integrated attention. To address this gap, this review synthesizes current understanding of ferroptosis-metabolism coupling in RCC, with the aim of bridging fundamental cell biology and clinical oncology. We first outline the overarching biochemical mechanisms that govern ferroptosis in renal tumors. We then examine subtype-specific metabolic vulnerabilities, with particular emphasis on the contrast between the lipid-storing phenotype of ccRCC and the mitochondrial dysfunction characteristic of chRCC. Finally, we discuss key translational challenges, including tissue specificity, renal safety, and the development of predictive biomarkers. Through this integrated perspective, we aim to provide a conceptual framework for evaluating ferroptosis as a future metabolically targeted therapeutic strategy in RCC.

## 2 Materials and methods

### 2.1 Study design and reporting framework

The design for this research was that of a structured narrative review which included systematic aspects and was performed according to the PRISMA 2020 (27). The goal of this review was to combine mechanistic, translational and emerging clinical data regarding metabolic reprogramming and ferroptosis in renal cell carcinoma (with an emphasis on chromophobe renal cell carcinoma). Due to the considerable variability in study design, including *in vitro* studies, *in vivo* models, and very limited clinical data, a qualitative integrated review was employed, rather than a quantitative meta-analysis.

### 2.2 Information sources and database selection

PubMed/MEDLINE(Medical Literature Analysis and Retrieval System Online) [https://pubmed.ncbi.nlm.nih.gov/], Web of Science(Core Collection) [https://www.webofscience.com] and Scopus [https://www.scopus.com] were used for the majority of this search because they are well-indexed for all types of biomedical, translational, and basic science literature that could be related to renal cancer biology and ferroptosis. In an effort to make sure no important studies were missed, we manually examined the reference lists of many key review articles and highly cited primary studies. We did not include any unpublished studies nor studies from pre-print servers to remain consistent with the standards of peer-reviewed literature.

### 2.3 Search strategy and keyword development

The development of the search strategy was a dynamic iterative process to identify studies that relate to renal cell carcinoma metabolism as well as chromophobe renal cell carcinoma and the ferroptosis related pathway(s). Where applicable, both free text terms (terms entered by users) and database-specific controlled vocabulary were utilized. Core search concepts for renal cell carcinoma, chromophobe renal cell carcinoma, cancer metabolism, mitochondrial dysfunction, glutathione metabolism, redox homeostasis, iron metabolism, lipid peroxidation, and ferroptosis were combined using Boolean operators (AND/OR/etc.) to combine disease-specific terms with metabolic/ferroptosis-related terms. Search strings for each database were modified to accommodate the varying ways databases index/syntax.

### 2.4 Search limits and filters

To ensure that researchers could accurately interpret the methodology used in the articles, searches were restricted to articles written in the English language. There is no specific minimum date for publication to be eligible; however, when synthesizing data, preference was given to studies that have been published over the past 15 years, as this represents significant advancements in both metabolomics and regulated cell death research, as well as, the classification of renal cancers. Articles that were eligible for inclusion were original research articles, mechanistic studies, translational investigations and selected clinical studies. Excluded from consideration were editorials, abstracts of conferences and other non-peer reviewed commentary.

### 2.5 Eligibility criteria

Studies were selected based on predefined inclusion and exclusion criteria that focused on relevance to renal cell carcinoma metabolism and ferroptosis related mechanisms. These criteria were established prior to screening and are summarized in **Table 1**.

**Table 1:** Inclusion and exclusion criteria for study selection.

Inclusion criteria	Exclusion criteria
Studies involving renal cell carcinoma, including chromophobe subtype	Studies not involving renal or kidney derived malignancies
Experimental, translational, or clinical studies addressing metabolism, redox biology, iron metabolism, or ferroptosis	Articles focused solely on unrelated cell death mechanisms without metabolic relevance
In vitro or in vivo models relevant to renal cancer biology	Non peer reviewed articles, editorials, letters, or conference abstracts
Studies published in English	Studies lacking sufficient methodological detail
Human, animal, or cell based research	Purely descriptive pathology reports without mechanistic or metabolic insight

### 2.6 Study selection process

All database search results were imported to reference management software and duplicates were eliminated. Titles and abstracts were evaluated independently for relevance to the objective of the review. Studies that clearly did not meet inclusion criteria were removed from further consideration. The full text versions of all remaining studies were retrieved and evaluated as to whether they met the final inclusion criteria. Any uncertainty about eligibility was resolved by reviewing the study objectives, methodology, and whether it relates to either ferroptosis or metabolic pathways in renal cell carcinoma.

### 2.7 PRISMA flow narrative

**Figure 1:**
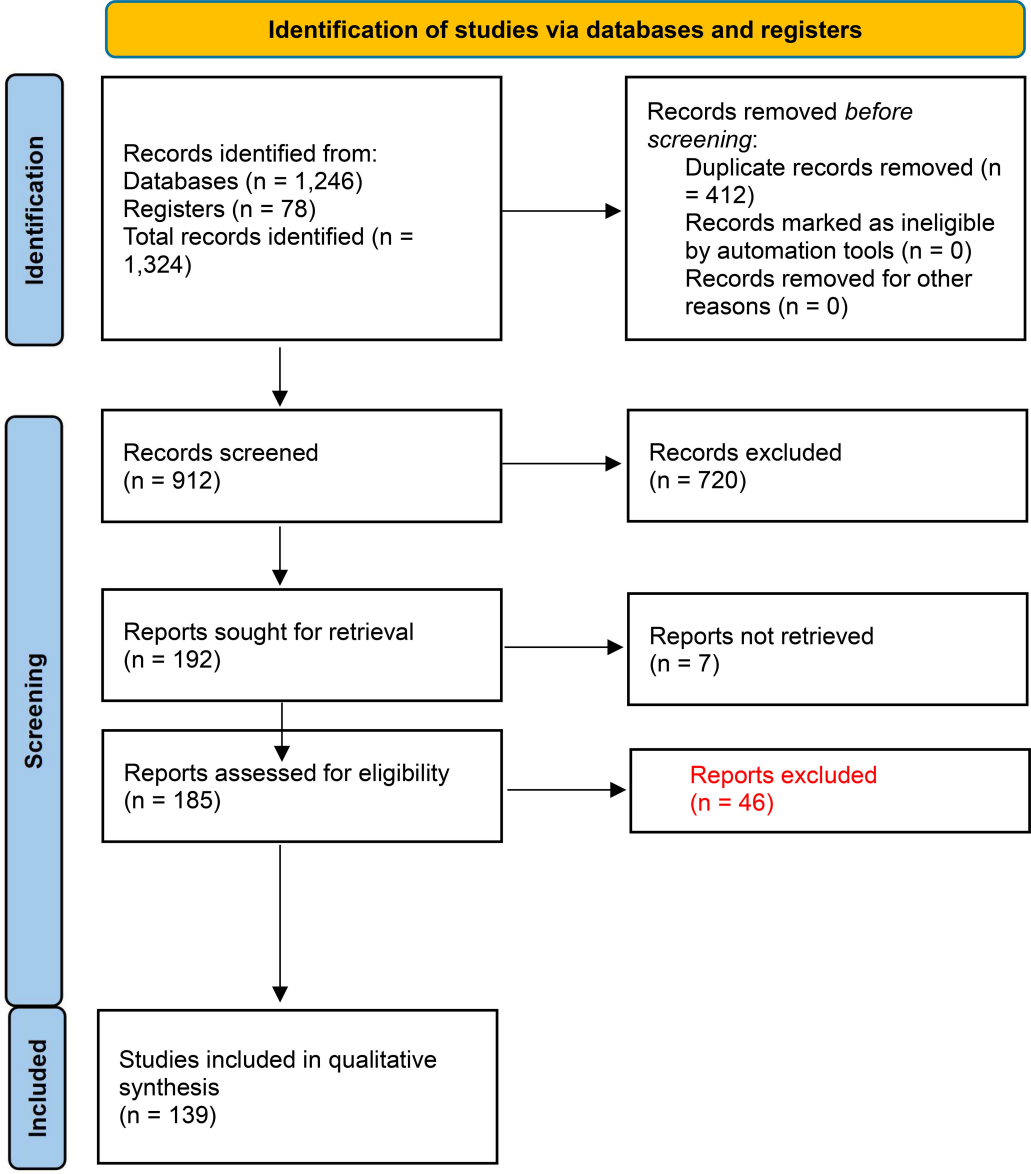
PRISMA 2020 flow diagram of study selection.

A total of 1,324 records were initially identified, including 1,246 records from database searches and 78 records from registers. After 412 duplicate records were removed, 912 records were screened by title and abstract for relevance to renal cell carcinoma, chromophobe renal cell carcinoma, metabolism, redox biology, iron metabolism, lipid peroxidation, and ferroptosis. Of these, 720 records were excluded because they were unrelated to renal or kidney-derived malignancies, did not address relevant metabolic or ferroptosis-related mechanisms, or represented non-peer-reviewed materials. A total of 192 reports were sought for retrieval, of which 7 were not retrieved. The remaining 185 reports were assessed according to the predefined inclusion and exclusion criteria. During full-text assessment, 46 reports were excluded because they lacked sufficient mechanistic detail, were not applicable to renal cancer biology, or did not provide relevant information on metabolism, redox regulation, iron handling, mitochondrial dysfunction, lipid peroxidation, or ferroptosis. Ultimately, 139 studies were retained for qualitative synthesis in this review. The PRISMA flow diagram has been revised accordingly to ensure that the numbers reported in the figure are consistent with the methodological narrative and the final reference list. As shown in **Figure 1**.

### 2.8 Data extraction and qualitative synthesis

Data from all studies that were reviewed were used to extract data regarding the design of the study, the type of experimental model or patient population examined in the study, whether the renal cell carcinoma (RCC) subtype was identified, the metabolic pathway being studied, the mechanism of ferroptosis, and the main results of the study. A focus was placed on any data that linked the mechanisms of mitochondria dysfunction, glutathione metabolism, iron handling and lipid peroxides. Findings were organized by theme rather than aggregated quantitatively to look for common mechanisms and patterns among different experimental models. Organizing the findings in this manner allowed diverse lines of evidence to be synthesized into a logical conceptual framework.

## 3 Mechanisms of ferroptosis-metabolism coupling in renal cell carcinoma

### 3.1 Lipid remodeling and membrane susceptibility

**Figure 2:**
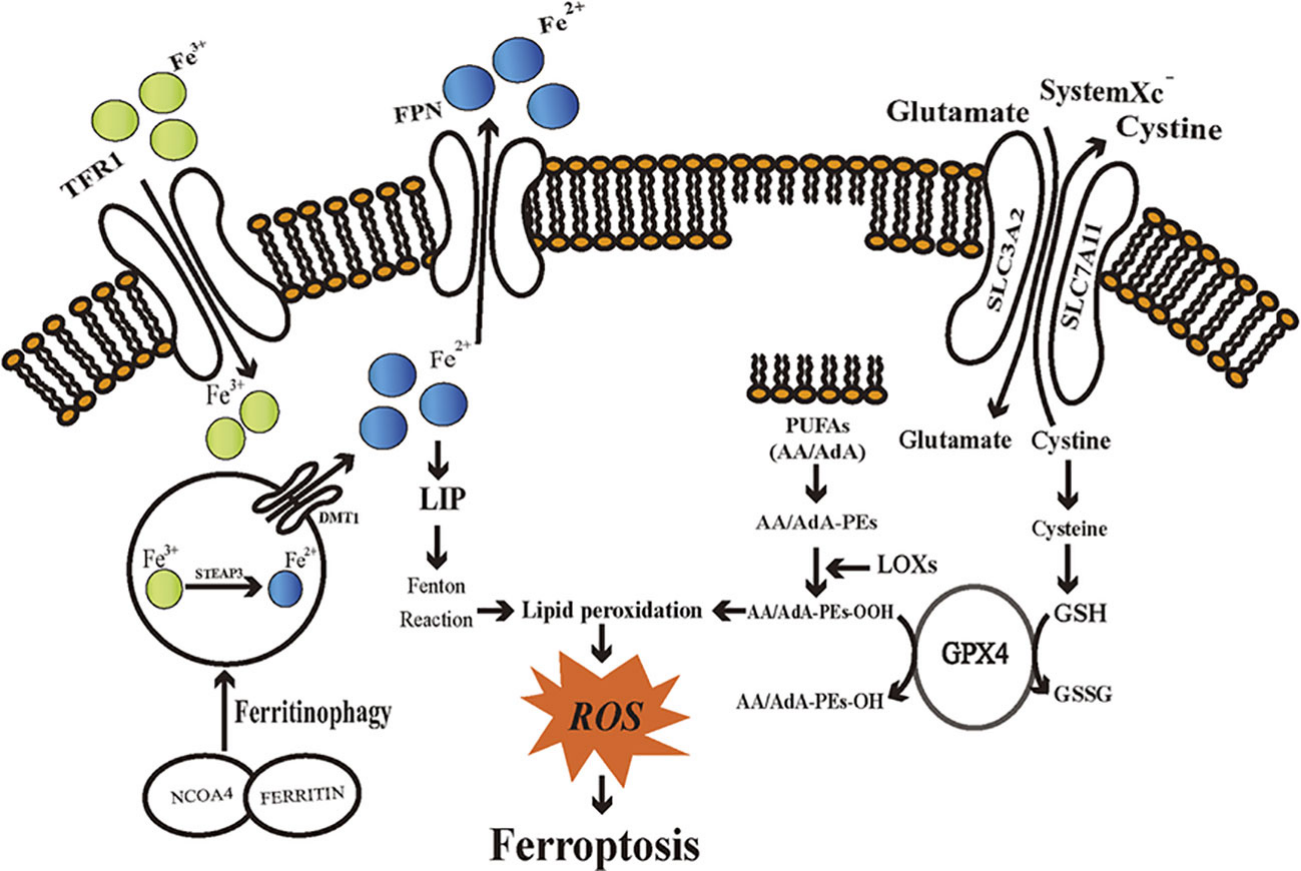
Schematic diagram of regulatory mechanisms of ferroptosis. Iron metabolic pathways, SystemXc ^-^/glutathione axis, and lipid peroxidation engage in the regulation of ferroptosis. TFR1, transferrin receptor 1; FPN, ferroportin; DMT1, divalent metal transporter 1; STEAP3, six-transmembrane epithelial antigens of the prostate 3; NCOA4, Nuclear receptor coactivator 4; LIP, labile iron pool; ROS, reactive oxygen species; SLC3A2, Solute Carrier Family 3 Member 2; SLC7A11, Solute Carrier Family 7 Member 11; PUFAs, polyunsaturated fatty acids; AA/AdA, arachidonic acid/adrenic acid (AdA); AA/AdA-PEs, AA/AdA-phosphatidylethanolamine (PE); AA/AdA-PEs-OH, AA/AdA-PEs-alcohols; AA/AdA-PEs-OOH, AA/AdA-PEs-hydroperoxides; GPX4, Glutathione peroxidases 4; GSH, glutathione; GSSG, glutathione disulfide; LOXs, Lipoxygenases (28).

**Figure 3:**
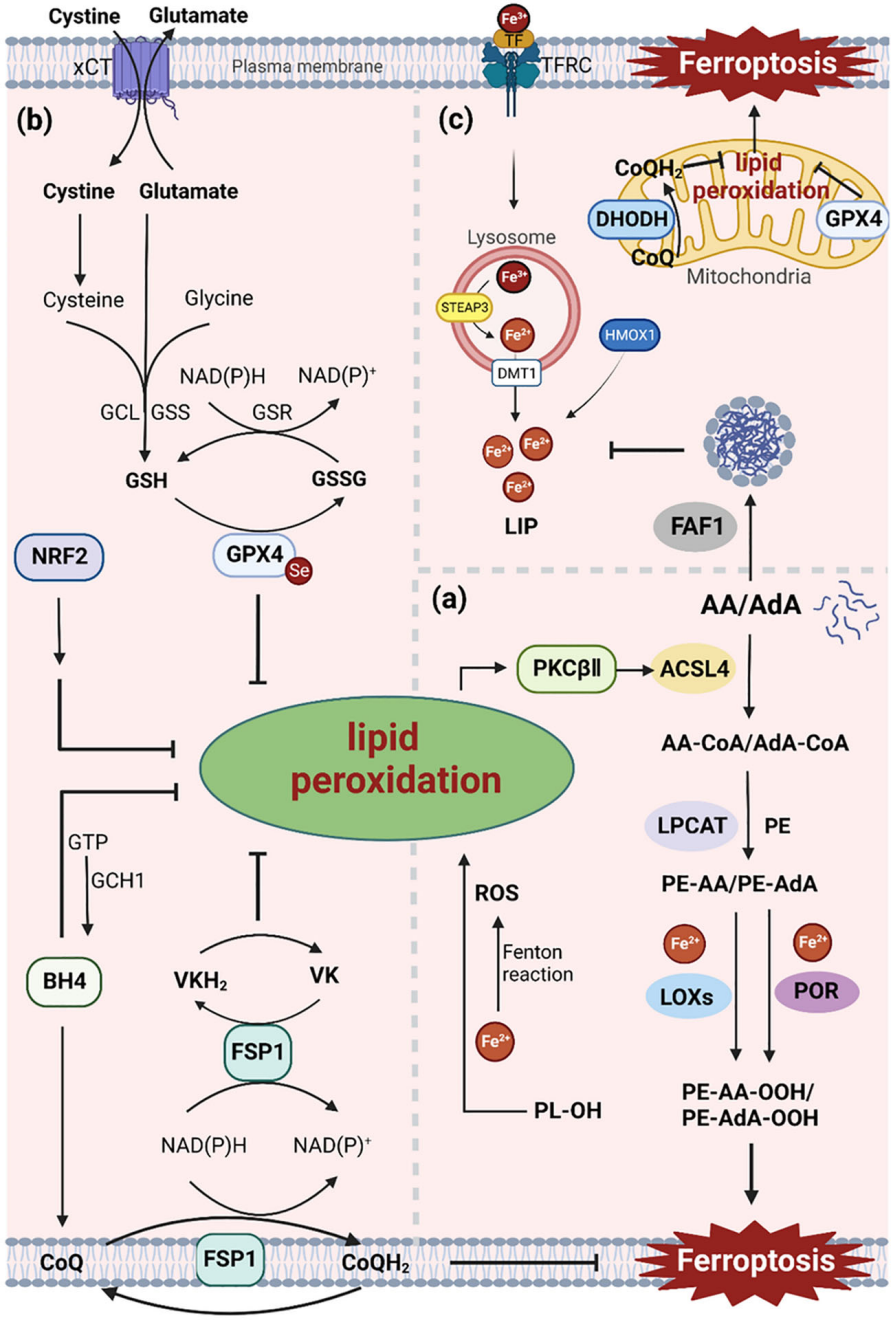
Mechanisms of ferroptosis and against lipid peroxidation. A schematic chart showing that ferroptosis is executed by lipid peroxidation, a process dependent on PUFAs (e.g., AA and AdA), metabolites reactive oxygen species, and the metallic element iron. **(a)** Mechanisms of ferroptosis. As key catalytic factors of ferroptosis, ACSL4 and LPCAT3 catalyze the production of AA-PE/AdA-PE. PKCβII can act as a lipid peroxidation sensor, and further phosphorylation of ACSL4 by activated PKCβII can amplify the effect of lipid peroxidation. The occurrence of lipid peroxidation occurs both enzymatically and non-enzymatically. LOXs and POR can catalyze enzymatic lipid peroxidation in a Fe²^+^-dependent manner. Non-enzymatic lipid peroxidation is a free radical-driven chain reaction, and ROS generated by the iron-dependent Fenton reaction can trigger the peroxidation of polyunsaturated fatty acids. **(b)** Antioxidant mechanisms against lipid peroxidation. GPX4 can prevent lipid peroxidation by reducing PL-OOH to nontoxic PL-OH. Glutathione is a cofactor of GPX4, and xCT can mediate the uptake of cystine required for GSH to synthesize GPX4. FSP1 can inhibit lipid peroxidation by reducing oxidized CoQ to CoQH2 in a GSH-independent manner. FSP1-dependent non-canonical vitamin K cycle is a ferroptosis suppressor to protect potently cells from lipid peroxidation. BH4 is an effective antioxidant against lipid peroxidation in a GPX4 and FSP1 independent manner. In addition, as a key transcription factor of cellular antioxidant response, Nrf2 can block lipid peroxidation by upregulating various ferroptosis inhibitory molecules. **(c)** Iron metabolism and antioxidant mechanisms of ferroptosis. Transferrin and TFRC are involved in Fe³^+^ transport. STEAP3 mediates the reduction of Fe³^+^ to Fe²^+^ and DMT1 mediates the transport of Fe²^+^ to the cytoplasm LIP, thereby triggering lipid peroxidation. The overexpression of HMOX1 increases LIP and promotes ferroptosis. Mitochondrial DHODH prevents lipid peroxidation by reducing CoQ to CoQH2, which is parallel to antioxidant mechanisms of mitochondrial GPX4. A newly discovered ferroptosis Blocking molecule, FAF1, sequesters the free PUFAs into a spherical structure to block the contact of PUFAs with iron to prevent ferroptosis. (GPX4, glutathione peroxidase 4; Se, Selenium; FSP1, ferroptosis inhibitory protein 1; DHODH, dihydroorotate dehydrogenase; FAF1, Fas-associated factor 1; LIP, labile iron pool; TF, transferrin; TFRC, transferrin receptor; DMT1, divalent metal transporter 1; HMOX1, heme oxygenase 1; STEAP3, six-transmembrane epithelial antigen of prostate 3; ROS, reactive oxygen species; LOXs, lipoxygenases; POR, cytochrome P450 oxidoreductase; AA, arachidonoyl; AdA, adrenoyl; ACSL4 acyl-CoA, synthetase long-chain family member 4; PEs, phosphatidylethanolamines; LPCAT3, lysophosphatidylcholine acyltransferase 3; PKCβII, protein kinase C-βII, isoform; VK, vitamin K; VKH2, vitamin K hydroquinone; BH4, tetrahydrobiopterin; xCT, cystine-glutamate transporter; GSH, reduced glutathione; GSS, Glutathione synthetase; GSR, Glutathione Reductase; GCL, glutamate-cysteineligase; GSSG, oxidized glutathione; GCH1, GTP-dependent cyclohydrolase 1; GTP, Guanosine triphosphate; Nrf2, nuclear factor erythroid 2-related factor 2; CoQ10, coenzyme Q10; CoQ, ubiquinone; CoQH2, ubiquinol; PL-OH, Phospholipid alcohol; PE-AA-OOH, phosphatidylethanolamine-arachidonoyl-hydroperoxide; PE-AdA-OOH, phosphatidylethanolamine-adrenoyl-hydroperoxide) (29).

**Figure 4:**
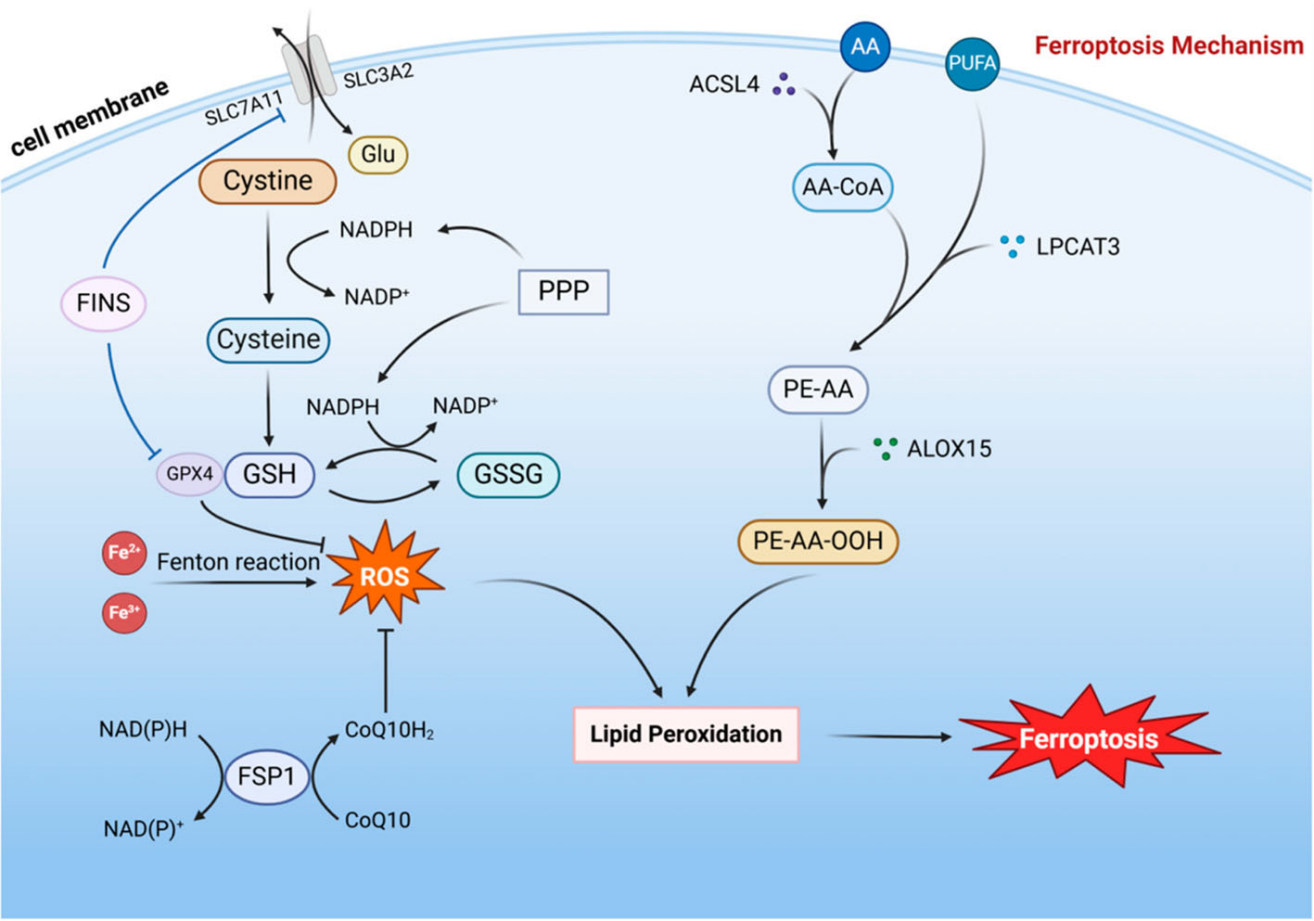
Ferroptosis mechanism: Ferroptosis is directly triggered by lipid peroxides, with iron ions catalyzing the Fenton reaction to generate reactive oxygen species (ROS) that lead to the formation of lipid peroxides. Polyunsaturated fatty acids (PUFAs) and arachidonic acid (AA) in the cell membrane can undergo a series of catalytic reactions to form peroxidized lipids, ultimately resulting in ferroptosis. Glutathione peroxidase 4 (GPX4) plays a pivotal role in inhibiting ferroptosis by consuming glutathione (GSH) to scavenge ROS. The Xc-system facilitates GSH synthesis by transporting glutamate and cystine, thereby suppressing ferroptosis. Ferroptosis inducers (FINs) promote ferroptosis by inhibiting both the Xc-system and GPX4. Ubiquinone (CoQ10), under the catalysis of ferroptosis suppressor protein 1 (FSP1), can be converted into its reduced form, CoQ10H2, which scavenges ROS and thus inhibits ferroptosis. (Abbreviations: SLC7A11, solute carrier family 7 member 11; SLC3A2, solute carrier family 3 member 2; PPP, pentose phosphate pathway; ACSL4, acyl-CoA synthetase long-chain family member 4; LPCAT3, lysophosphatidylcholine acyltransferase 3; ALOX15, arachidonate 15-lipoxygenase) (30).

To facilitate an intuitive understanding of the complex ferroptosis machinery in renal cell carcinoma, the reader is guided through three integrated schematic figures. **Figure 2** provides a global overview of the core ferroptosis regulatory network, including iron metabolic pathways, the System Xc^-^/glutathione/GPX4 axis, and lipid peroxidation events. **Figure 3** delves deeper into the enzymatic and non-enzymatic mechanisms of lipid peroxidation, along with the parallel antioxidant defense systems (GPX4-dependent, FSP1-dependent, and others) that determine ferroptosis susceptibility. **Figure 4** synthesizes these concepts into a therapeutic framework, illustrating how pharmacological targeting of the Xc^-^ system, GPX4, FSP1, or mitochondrial and iron handling pathways can lower the threshold for ferroptotic cell death. Collectively, these figures form a step-by-step visual roadmap that supports the detailed mechanistic discussions in the following subsections.

Metabolic reprogramming across different subtypes of renal cell carcinoma (RCC) frequently involves distinct alterations in lipid metabolism. Instead of functioning solely as an energy reserve, these metabolic shifts influence membrane composition and oxidative stress responses (31). For example, clear cell RCC (ccRCC) is particularly characterized by the upregulation of neutral lipid synthesis and subsequent storage in intracellular lipid droplets. This compartmentalization process sequesters polyunsaturated fatty acids (PUFAs) away from cellular membranes. By restricting the availability of free PUFAs, lipid droplets limit their incorporation into phospholipids, a process that is associated with reduced structural susceptibility to lipid peroxidation (32, 33).

When fatty acids bypass lipid droplet storage, they undergo enzymatic desaturation and elongation before integrating into cell membranes. The specific biochemical composition of these membranes strongly influences the cellular potential to undergo ferroptosis. Specifically, phospholipids containing long-chain PUFAs, such as arachidonic acid and adrenic acid, are known substrates for lipid peroxidation (34). Experimental models suggest that tumor cells utilize distinct lipid remodeling enzymes, including acyl-CoA synthetases and lysophosphatidylcholine acyltransferases, to mediate the integration of these PUFAs into the lipid bilayer. This remodeling process appears to contribute to how certain RCC cells maintain membrane fluidity under oxidative stress (35). Because lipid remodeling strategies vary significantly among ccRCC, papillary RCC, and chromophobe RCC (chRCC), the quantitative distribution of PUFA-containing phospholipids likely establishes different baseline vulnerabilities to ferroptotic stimuli across the RCC spectrum.

However, the accumulation of oxidizable PUFAs is generally insufficient to initiate ferroptosis independently. The progression of this cell death pathway relies on a continuous source of oxidative stress. In several RCC variants, this oxidative pressure is frequently associated with mitochondrial dysfunction. The quantity and functional integrity of mitochondria in renal tumors are often compromised by defective biogenesis or impaired quality control mechanisms, such as disrupted mitophagy (36, 37) Consequently, certain RCC subtypes, particularly chRCC, tend to accumulate morphologically abnormal mitochondria. Functional impairments within the electron transport chain can cause continuous electron leakage. These electrons subsequently react with oxygen to form superoxide radicals and reactive oxygen species (ROS). This sustained ROS generation provides an oxidative environment that contributes to the peroxidation of membrane-bound PUFAs (38). Thus, the retention of dysfunctional mitochondria generally correlates with an increased cellular dependency on antioxidant systems to preserve membrane integrity.

The biochemical interaction between lipid substrate availability and oxidative stress is spatially regulated at mitochondria-associated endoplasmic reticulum (ER) membranes. The ER synthesizes and remodels membrane phospholipids, while adjacent mitochondria generate ROS and participate in intracellular iron metabolism (39, 40). This physical proximity facilitates direct signaling and metabolic exchange. In the context of RCC models exhibiting baseline mitochondrial defects, local ROS production may be amplified at these specific organelle contact sites. Consequently, newly synthesized PUFAs emerging from the ER are likely exposed to localized oxidative stress (41). This structural and functional coupling supports the notion that ferroptosis susceptibility in RCC is not a uniform cellular response. Rather, it represents a highly localized biochemical vulnerability. This vulnerability is largely influenced by the spatial intersection of active lipid synthesis and defective mitochondrial respiration. These interactions between lipid remodeling, mitochondrial ROS generation, antioxidant buffering, and iron handling are summarized in **Figure 2**, which provides a general framework for the subsequent discussion of RCC subtype-specific ferroptosis vulnerabilities.

### 3.2 Redox homeostasis and the GSH/GPX4 defense axis

The continuous generation of reactive oxygen species (ROS) and the presence of oxidizable membrane lipids necessitate robust antioxidant defense mechanisms to prevent cellular death. Glutathione (GSH), a tripeptide composed of glutamate, cysteine, and glycine, serves as a primary intracellular antioxidant in this context (42, 43). In many cancer models, including specific subtypes of renal cell carcinoma (RCC), the biosynthesis of GSH is restricted by the intracellular availability of cysteine (44, 45). To maintain adequate cysteine levels, RCC cells frequently upregulate the expression of the cystine/glutamate antiporter, known as system xCT (SLC7A11) (46, 47). This transporter functions by importing extracellular cystine in exchange for intracellular glutamate. Once inside the cell, cystine is reduced to cysteine. This reduction process consumes cellular reducing equivalents, particularly NADPH. Consequently, the functional dependency on system xCT links cellular redox defense mechanisms directly to central carbon metabolism (48). Furthermore, because system xCT actively exports glutamate, its chronic activation alters intracellular glutamate pools. This alteration influences parallel metabolic pathways, including anaplerosis and nitrogen metabolism, suggesting a complex metabolic trade-off in cancer cells attempting to survive under oxidative stress (49).

The functional significance of GSH in preventing ferroptosis is primarily mediated through the activity of glutathione peroxidase 4 (GPX4). GPX4 utilizes GSH as an essential cofactor to reduce toxic phospholipid hydroperoxides into stable, non-toxic lipid alcohols (50). By neutralizing these lipid peroxides, GPX4 intercepts the propagation of free radical chain reactions within the cellular membrane. A reduction in GSH availability or the direct impairment of GPX4 activity generally leads to the accumulation of un-repaired lipid hydroperoxides. When this accumulation surpasses the cellular detoxification capacity, it initiates the execution phase of ferroptosis (51). In addition to the primary GSH/GPX4 axis, certain tumor cells utilize alternative, GSH-independent defense systems to modulate ferroptosis sensitivity. For example, ferroptosis suppressor protein 1 (FSP1) localizes to the cellular membrane to regenerate ubiquinol (CoQ10H2), a process that intercepts lipid radicals independently of GPX4 activity (52, 53). The coordinated function of these parallel defense systems illustrates that ferroptosis resistance relies on an integrated network of redox-regulating pathways.

Within the spectrum of renal malignancies, the reliance on these specific redox defense systems exhibits significant subtype-dependent variation. In clear cell RCC (ccRCC), metabolic adaptations driven by hypoxia-inducible factor (HIF) signaling actively reshape central carbon metabolism. This molecular remodeling is associated with altered NADPH production capacity, which subsequently influences the efficiency of GSH recycling and overall antioxidant capacity (54, 55). Conversely, chromophobe RCC (chRCC) models present a distinct redox phenotype. As discussed previously, chRCC cells frequently exhibit severe structural abnormalities in mitochondria and impaired oxidative metabolism. These organelle defects contribute to elevated basal levels of intracellular ROS (56, 57). Because of this sustained oxidative pressure, chRCC cells appear to depend heavily on the continuous availability of GSH and robust GPX4 activity to preserve membrane integrity (58). Therefore, the therapeutic disruption of cystine supply or GPX4 function is hypothesized to induce selective vulnerability in specific RCC subtypes. This subtype-specific dependency underscores the necessity of evaluating baseline redox stress when investigating ferroptosis-targeted strategies in renal cancer (59–61).

### 3.3 Iron dysregulation and the initiation of lipid peroxidation

Normal renal tubular epithelial cells maintain iron homeostasis through a tightly coordinated system of import, storage, and export to prevent intracellular toxicity. Following transferrin receptor 1 (TFR1)-mediated endocytosis, ferrous iron is exported from endosomes into the cytosol primarily by divalent metal transporter 1 (DMT1) and either directed to mitochondria for utilization, safely sequestered in ferritin nanocages for storage, or exported across the basolateral membrane by ferroportin (FPN1). This multilayered defense normally prevents accumulation of redox-active iron that could trigger Fenton-driven lipid peroxidation (62).

During malignant transformation in RCC, however, these regulatory controls are profoundly rewired. RCC cells upregulate iron acquisition through TFR1 overexpression, expanding the labile iron pool (LIP) to meet biosynthetic and metabolic demands (63). Simultaneously, iron storage is dynamically regulated via NCOA4-mediated ferritinophagy, which releases stored iron and has been linked to unfavorable prognosis and defective immune infiltration in clear cell RCC (64, 65). Cancer cells also modulate iron export; hepcidin-mediated suppression of FPN1 further promotes intracellular iron retention (62). The resulting expansion of the LIP, combined with baseline oxidative stress, lowers the threshold for iron-catalyzed lipid peroxidation.

In chromophobe RCC (chRCC), the convergence of abundant catalytic iron and continuous mitochondrial ROS generation—driven by impaired iron-sulfur cluster assembly in dysfunctional mitochondria—primes cells for ferroptosis (66). Yet these same cells simultaneously upregulate glutathione-dependent antioxidant defenses to maintain viability, creating a state of contextual vulnerability. Thus, pharmacological agents that disrupt cystine import or GPX4 function overwhelm compensatory capacity and tip the balance toward ferroptotic death (67, 68).

Renal epithelial cells are continuously exposed to circulating transferrin-bound iron. This iron is necessary to support high rates of mitochondrial respiration and the synthesis of iron-sulfur (Fe-S) clusters (62). During malignant transformation, renal cell carcinoma (RCC) frequently exhibits altered iron homeostasis. Clinical and experimental observations indicate that early-stage RCC tumorigenesis is typically associated with intracellular iron accumulation (63). Cancer cells generally upregulate iron acquisition through transferrin receptor-1 (TfR1)-mediated endocytosis. Following endocytic processing, ferrous iron is released into the cytoplasm to form the labile iron pool (LIP). While this non-heme iron supports essential biosynthetic pathways, it is highly redox-active. An expanded LIP directly contributes to the generation of reactive oxygen species (ROS) through the Fenton reaction. This specific iron-catalyzed reaction provides the necessary chemical activity to initiate the peroxidation of polyunsaturated phospholipids (69).

To mitigate the oxidative risk associated with the LIP, cells store excess iron within ferritin complexes in a redox-inactive state. However, intracellular iron storage is a dynamic process that is frequently reprogrammed in tumors. Cancer cells can mobilize stored iron through ferritinophagy, a selective autophagic process mediated by the nuclear receptor coactivator 4 (NCOA4) (70, 71). NCOA4 binds to ferritin and targets it for lysosomal degradation, which subsequently releases free iron back into the cytoplasm. In various cancer models, enhanced NCOA4-mediated ferritinophagy significantly increases the size of the LIP (64, 65, 72). While this mobilized iron supports tumor proliferation, it simultaneously increases cellular vulnerability to iron-driven lipid peroxidation (65, 72). Consequently, the regulatory balance between ferritin storage and NCOA4-mediated degradation represents a critical determinant of ferroptosis susceptibility.

The specific mechanisms of iron dysregulation exhibit notable variation among distinct RCC subtypes. In clear cell RCC (ccRCC), iron metabolism appears to intersect closely with hypoxia-inducible factor (HIF) signaling. For instance, the inhibition of specific Fe-S cluster assembly proteins, such as ISCA2, has been shown to decrease HIF levels and sensitize ccRCC cells to ferroptosis (73). This functional connection suggests that ccRCC utilizes specific iron-dependent protein networks to maintain its pseudo-hypoxic phenotype and resist oxidative death. Alternatively, chromophobe RCC (chRCC) presents a different biochemical dynamic. Because chRCC is characterized by severe baseline mitochondrial dysfunction, the cells already experience elevated basal ROS production (74). In this specific context, structural defects in mitochondria often impair normal Fe-S cluster assembly and disrupt intracellular iron trafficking (75, 76). This disruption can lead to an accumulation of reactive iron. The combination of abundant catalytic iron and continuous mitochondrial electron leakage creates a biochemical environment that facilitates extensive lipid peroxidation. Therefore, assessing subtype-specific iron handling is essential for understanding how renal tumors ultimately undergo ferroptotic cell death. Accordingly, **Table 2** is used as a translational stratification framework linking ferroptosis-related vulnerabilities to candidate biomarker domains and future therapeutic prioritization, while **Figure 3** provides a pathway-level overview of how mitochondrial dysfunction, redox buffering, lipid remodeling, and iron handling converge to shape ferroptosis susceptibility in RCC.

**Table 2:** Metabolic vulnerabilities and candidate translational stratification domains across renal cell carcinoma subtypes, with emphasis on chromophobe disease.

RCC subtype	Dominant metabolic features	Ferroptosis relevant liabilities	Translational implications and candidate biomarker domains
Clear cell RCC (77)	Hypoxia signaling driven remodeling, altered glucose utilization, prominent lipid storage phenotype, variable mitochondrial activity	Context dependent susceptibility shaped by membrane lipid composition, oxidative pressure, and antioxidant capacity	Lipid remodeling signatures, oxidative stress markers, iron handling markers, expression of antioxidant defense pathways
Papillary RCC (78)	Heterogeneous metabolic states, variable mitochondrial remodeling and nutrient utilization patterns	Subset specific vulnerability when redox dependence or iron dysregulation is pronounced	Metabolic subtype stratification, mitochondrial stress indicators, redox pathway activity signatures
Chromophobe RCC (10)	Frequent mitochondrial accumulation with structural and functional abnormalities, oxidative metabolism perturbation, elevated redox pressure	Potential dependency on glutathione dependent lipid peroxide detoxification, susceptibility under iron mediated oxidative stress, limited buffering under additional redox disruption	Mitochondrial content and stress markers, glutathione pathway activity, iron trafficking and storage markers, lipid peroxidation related signatures

## 4 Subtype-specific metabolic vulnerabilities and ferroptosis sensitivity

Quantitative evidence across RCC subtypes highlights chromophobe RCC (chRCC) as a particularly ferroptosis-sensitive entity. Compared with normal kidney tissue, chRCC exhibits marked glutathione metabolic dependency and reliance on cystine import via SLC7A11, with hypersensitivity to ferroptosis induced by system Xc^-^ or GPX4 inhibition (67). Transcriptomic analyses across TCGA reveal that chRCC has the second-highest upregulation of FSP1 among all cancer types, and high FSP1 expression correlates with significantly poorer patient outcomes; functional validation shows that FSP1 inhibitor monotherapy reduces chRCC tumor growth by 69% in xenograft models (68). In clear cell RCC (ccRCC), lipid droplet accumulation modulates ferroptosis susceptibility by sequestering polyunsaturated fatty acids, and therapeutic disruption of this storage phenotype enhances ferroptosis induction (79). These quantitative data collectively establish that RCC subtypes possess distinct, targetable ferroptosis vulnerabilities, with chRCC operating closest to its ferroptotic threshold.

### 4.1 The Pseudo-hypoxic and lipid-storing phenotype of ccRCC

Clear cell renal cell carcinoma (ccRCC) is characterized by a distinct pseudohypoxic metabolic phenotype, which is primarily associated with the constitutive activation of hypoxia-inducible factors, particularly HIF-2α. Rather than operating in isolation, this pathway is linked to extensive alterations in lipid metabolism and cellular rewiring (80, 81). Under these pseudohypoxic conditions, ccRCC cells frequently upregulate the expression of genes involved in fatty acid uptake and lipid storage. For instance, studies indicate that targeting oncogenic pathways, such as MYC, induces lipid droplet accumulation through the upregulation of hypoxia-inducible lipid droplet-associated protein (HILPDA) (82). Observations suggest that this accumulation of neutral lipids contributes to the maintenance of endoplasmic reticulum (ER) homeostasis, thereby supporting tumor cell survival and progression under stress (83).

In addition to neutral lipid storage, ccRCC often exhibits notable alterations in phospholipid biosynthesis pathways (84). Recent investigations have begun to map how specific molecules regulate this lipid balance to shape oxidative stress responses. For example, enzymes such as fatty acid desaturase 1 (FADS1) are implicated in modulating fatty acid desaturation and altering membrane lipid composition. Concurrently, specific G-protein coupled receptors, such as GPR1 and CMKLR1, appear to function in the signaling regulation of lipid remodeling and stress adaptation (85). By integrating these pathways, ccRCC cells can modify their lipid distribution. Through sequestering polyunsaturated fatty acids into lipid droplets or altering phospholipid chain composition, these adaptive processes may limit the availability of substrates required for lipid peroxidation, which supports the notion of ferroptosis evasion (79).

This specialized lipid phenotype also correlates with broader implications for the tumor microenvironment. Clinical analyses indicate that the lipid metabolism profile of ccRCC is associated with patient survival outcomes and influences the state of intratumoral CD8+ T cells (86). These observations suggest that the lipid-storing phenotype of ccRCC may create exploitable metabolic liabilities. In principle, therapeutic approaches that disrupt lipid droplet storage or promote the incorporation of polyunsaturated fatty acids into membrane phospholipids could increase the susceptibility of ccRCC cells to ferroptosis-inducing agents (79).

### 4.2 Severe mitochondrial dysfunction and redox dependency in chRCC

Unlike the hypoxia-driven and lipid-storing phenotype frequently observed in clear cell RCC, chromophobe renal cell carcinoma (chRCC) exhibits a fundamentally different metabolic profile. A characteristic feature of chRCC is the intracellular accumulation of morphologically abnormal mitochondria (10). Histological and ultrastructural analyses frequently demonstrate this dense mitochondrial packing, which distinguishes chRCC from other renal tumor variants (11). However, this accumulation is generally not indicative of enhanced oxidative phosphorylation. Rather, it appears to represent a cellular response to underlying defects within the respiratory chain. Recent mechanistic studies highlight specific impairments in mitochondrial complex I in these tumor cells (87). Defects within complex I can compromise efficient electron transport, leading to electron leakage. This leakage is thought to promote the basal generation of superoxide and other reactive oxygen species (ROS). Consequently, chRCC cells typically operate under a state of chronic oxidative stress, a condition that likely influences their metabolic dependencies and survival strategies.

Although experimentally tractable models of chRCC remain limited, the few available chRCC-derived models, including UOK-derived systems used in the recent literature, have provided important evidence for these metabolic vulnerabilities. In particular, studies of chRCC models have suggested that this subtype may display a marked dependency on glutathione metabolism and cystine import through SLC7A11, which can increase sensitivity to ferroptosis-inducing perturbations (67). Broader analyses of hereditary and sporadic RCC metabolism also support the concept that chRCC should not be interpreted simply as a variant of ccRCC, because its biology is more closely linked to mitochondrial dysfunction, oxidative stress adaptation, and altered redox buffering (88). In addition, recent ferroptosis-focused studies in RCC and chRCC models have further implicated lipid peroxide detoxification pathways, including FSP1, as compensatory survival mechanisms that may shape therapeutic response (68, 89). These model-based findings strengthen the rationale for targeting ferroptosis-related dependencies in chRCC, while also emphasizing that conclusions from the currently available models should be validated in additional patient-derived and *in vivo* systems.

The structural and functional mitochondrial impairments in chRCC may also influence intracellular iron homeostasis, potentially amplifying oxidative vulnerability. Mitochondria serve as the primary compartments for the biogenesis of iron-sulfur (Fe-S) clusters, which act as essential cofactors for numerous metabolic enzymes (66). When mitochondrial integrity and respiratory function are compromised, the assembly and export of Fe-S clusters are frequently disrupted. This disruption is associated with the inappropriate accumulation of redox-active iron within the mitochondrial matrix and adjacent cellular compartments (90). The presence of labile iron in an oxidative environment may facilitate the Fenton reaction. This chemical process catalyzes the conversion of endogenous hydrogen peroxide into highly reactive hydroxyl radicals, which can act as initiators of membrane damage (91).Therefore, the convergence of complex I defects and localized iron dysregulation is associated with a biochemical environment in chRCC that may be conducive to lipid peroxidation.

To maintain viability under this elevated endogenous oxidative pressure, chRCC cells appear to depend heavily on robust antioxidant defense networks. This apparent reliance on redox buffering is associated with a potential therapeutic vulnerability (92). As the basal production of lipid peroxides is likely elevated by mitochondrial dysfunction, chRCC cell viability may be closely linked to pathways that neutralize these lipid species. While the glutathione-dependent GPX4 axis provides a baseline defense, recent experimental evidence suggests that alternative ferroptosis suppressor pathways may also contribute to chRCC survival. Notably, the ferroptosis suppressor protein 1 (FSP1) system has been identified as a parallel defense mechanism in this context (68). Pharmacological inhibition of FSP1 has been reported to induce ferroptosis in certain chRCC models, highlighting a potential role for this lipid radical-scavenging enzyme. These findings support the notion that the unique mitochondrial and iron-handling features of chRCC may leave these cells with a limited capacity to buffer additional oxidative insults. Consequently, the targeted disruption of specific lipid peroxide detoxification pathways, such as FSP1 or GPX4, represents a potential biological strategy for further investigation in this RCC subtype.

### 4.3 Metabolic plasticity and adaptive resistance

The clinical management of certain RCC subtypes, particularly clear cell RCC (ccRCC), is often complicated by adaptive resistance to targeted therapies, such as tyrosine kinase inhibitors (TKIs) (92). Evidence from various solid tumor models suggests that cancer cells may survive therapeutic stress by entering a metabolically rewired persister state (93, 94). Lipidomic analyses in these broader cancer models indicate that this transition is frequently associated with expansion of the intracellular pool of polyunsaturated phospholipids (95). This accumulation of oxidizable lipids has been reported to correlate with enhanced baseline sensitivity to lipid peroxidation and, in some settings, increased susceptibility to ferroptotic damage.

To counteract this potential vulnerability, drug-tolerant cells appear to upregulate specific antioxidant defense mechanisms. For instance, stabilization of GPX4 by the deubiquitinase USP20 has been linked to TKI evasion across several cancer types (96). Although these molecular interactions require further validation in primary RCC tissues, they raise the possibility that TKI-resistant ccRCC subpopulations may exhibit altered ferroptosis susceptibility. Taken together, these observations support the notion that metabolic plasticity—particularly the balance between therapy-induced lipid remodeling and compensatory GPX4 stabilization—may influence the survival of persister cells.

Beyond cell-intrinsic adaptations, the metabolic composition of the tumor microenvironment (TME) may also shape therapeutic outcomes. Metabolic shifts within the RCC microenvironment during targeted or immune therapies have increasingly been recognized (97). In this context, emerging pan-cancer literature has proposed the concept of immunoferroptosis, describing a bidirectional metabolic crosstalk between tumor cells and the immune system (98). In certain experimental models, activated CD8+ T cells have been reported to secrete cytokines that impair tumor cell cystine uptake, a process associated with enhanced tumor ferroptosis (99).

Conversely, a metabolically hostile TME may induce lipid peroxidation in infiltrating immune cells, a process that appears to contribute to localized immunosuppression and tumor evasion (100). Taken together, observations from RCC and related cancer models raise the possibility that ferroptosis susceptibility in RCC is a dynamic state that may be modulated by prior systemic treatments and immune interactions (101). Consequently, targeting the acquired metabolic dependencies of drug-tolerant cells, or rationally combining ferroptosis modulators with established TKIs, may represent a therapeutic opportunity worthy of further investigation in selected RCC clinical settings.

## 5 Clinical translation, safety, and future outlook

### 5.1 Pharmacological targeting of metabolic dependencies

Current systemic therapies for renal cell carcinoma, including angiogenesis inhibitors and immune checkpoint blockade, primarily target extrinsic factors within the tumor microenvironment. While these regimens provide clinically relevant benefits for advanced clear cell RCC (ccRCC), they often exhibit limited efficacy in non-clear cell subtypes, such as chromophobe RCC (chRCC), which display distinct angiogenic and immune profiles. This clinical limitation highlights the necessity of addressing tumor-intrinsic metabolic vulnerabilities. Consequently, exploiting specific metabolic dependencies through the pharmacological induction of ferroptosis may represent a novel therapeutic opportunity, particularly for subtypes that are unresponsive to standard systemic regimens or have developed acquired resistance (such as tyrosine kinase inhibitor-tolerant states).

Pharmacological strategies to induce ferroptosis generally aim to disrupt the regulation of iron-dependent lipid peroxidation (102–104). One primary approach involves restricting cystine uptake or inhibiting glutathione peroxidase 4 (GPX4). This strategy is hypothesized to impair the cellular capacity to detoxify lipid peroxides. Such an approach may be particularly relevant in chRCC models characterized by elevated basal reactive oxygen species (ROS) production, where the antioxidant buffering system is already operating near maximum capacity. However, preclinical evidence suggests that cancer cells can dynamically upregulate alternative antioxidant pathways or utilize transsulfuration to maintain redox homeostasis (30, 105). Therefore, the efficacy of redox-targeted interventions will likely depend on defining subtype-specific metabolic bottlenecks rather than applying a universal blockade.

Several representative ferroptosis-targeting agents have been used in preclinical cancer studies to interrogate these pathways. System Xc− inhibitors, such as erastin and sulfasalazine, are commonly used to restrict cystine import and suppress glutathione synthesis, whereas GPX4-directed agents, including RSL3 and related compounds, directly impair lipid peroxide detoxification (106, 107). Additional strategies include iron-loading or iron-based nanomedicine approaches, as well as inhibition of compensatory ferroptosis suppressor pathways such as FSP1 (107, 108). However, most of these agents remain experimental or early translational tools, and none has yet established a defined clinical role as a ferroptosis inducer in RCC. Therefore, their relevance to RCC should be interpreted as a framework for trial development rather than as evidence of immediate clinical applicability.

Targeting tumor lipid metabolism provides another theoretical avenue to enhance peroxidation susceptibility (105). In ccRCC, which is classically defined by a pronounced lipid-storing phenotype, pharmacological inhibition of neutral lipid droplet formation is hypothesized to redirect free fatty acids toward membrane integration. This metabolic diversion may increase the fraction of polyunsaturated fatty acids (PUFAs) available within the phospholipid bilayer, potentially expanding the substrate pool for lipid peroxidation (109). Several specific approaches have been experimentally validated. First, lipid droplet-associated protein PLIN2 has been identified as a hub gene for ferroptosis in kidney renal clear cell carcinoma; its expression levels correlate with patient prognosis, making PLIN2 an effective new target for treatment (110). Second, stearoyl-CoA desaturase 1 (SCD1) — which converts saturated fatty acids to monounsaturated fatty acids — is consistently overexpressed in ccRCC; the pharmacological SCD1 inhibitor A939572 induces tumor-specific growth inhibition and cell death in ccRCC models (111). Third, genetic or pharmacological inhibition of fatty acid desaturase 1 (FADS1) reduces conversion of long-chain PUFAs, suppresses renal cancer cell proliferation, and induces cell cycle arrest, supporting FADS1 as a novel therapeutic target (112). Fourth, the natural flavonoid chrysin induces ferroptosis in RCC cells by suppressing the PI3K/Akt/GPX4 axis, as evidenced by increased ROS levels, Fe²^+^ accumulation, GSH depletion, and lipid peroxidation; notably, chrysin also enhances sunitinib sensitivity in RCC (113). These examples illustrate that targeting specific nodes in lipid metabolism — lipid droplet storage (PLIN2), fatty acid desaturation (SCD1, FADS1), and redox regulation (GPX4 pathway) — can effectively lower the ferroptosis threshold in RCC. Because lipid remodeling strategies and baseline membrane compositions vary significantly across the RCC spectrum, future studies should define how lipid-targeted therapies can be matched to specific histopathological or metabolic phenotypes.

Similarly, expanding the labile iron pool or exacerbating existing mitochondrial stress may lower the threshold for ferroptotic cell death (114). In chRCC, where structurally abnormal mitochondria generate substantial baseline oxidative pressure, relatively minor pharmacological increases in mitochondrial stress might exceed the compensatory limits of the tumor cells. This targeted intersection of iron dysregulation and mitochondrial stress supports the rationale for exploring ferroptosis induction in specific RCC variants.

Despite these mechanistic opportunities, translating these preclinical observations into clinical practice requires navigating significant safety boundaries. Broadly inhibiting redox defenses or inducing systemic iron dysregulation poses considerable toxicity risks to normal tissues, particularly given the kidney’s inherent physiological reliance on intact antioxidant buffering. Consequently, evaluating ferroptosis-based strategies necessitates a rigorous assessment of the therapeutic window. Future investigations must prioritize identifying subtype-selective vulnerabilities that minimize off-target effects. Furthermore, while combining ferroptosis inducers with standard therapies (e.g., targeted therapies or immunotherapies) offers potential synergistic benefits, it also introduces the risk of amplifying cumulative systemic toxicities, highlighting the need for highly context-dependent clinical trial designs.

### 5.2 Tissue specificity, off-target nephrotoxicity, and renal safety concerns

The clinical translation of ferroptosis-inducing therapies faces a significant challenge regarding tissue specificity, primarily due to the unique physiological characteristics of the normal kidney. Renal tubular epithelial cells possess a high density of mitochondria to support the energetic demands of continuous solute transport. This high metabolic activity generates substantial baseline oxidative stress. Consequently, normal renal tissue relies heavily on intact antioxidant defense systems, particularly the glutathione (GSH) and GPX4 axis, to maintain cellular viability. Recent studies in non-oncological renal pathologies, such as acute kidney injury (AKI), demonstrate that normal renal tubular epithelial cells are highly susceptible to ferroptosis. Pathological stressors that promote mitochondrial ROS production can rapidly trigger ferroptotic cell death in these epithelial populations (115–117). Furthermore, the identification of specific ferroptosis-related genes as critical biomarkers in AKI emphasizes the inherent vulnerability of the renal parenchyma to unchecked lipid peroxidation (118).

Because the normal kidney operates with a continuous requirement for lipid peroxide detoxification, systemic pharmacological induction of ferroptosis poses serious safety concerns. Experimental models of AKI indicate that the suppression or genetic disruption of GPX4 directly drives extensive renal ferroptosis. This disruption is not only associated with severe acute tissue damage but also appears to promote the progression from acute injury to chronic kidney disease (CKD) (119, 120). Therefore, therapeutic strategies that broadly inhibit cystine uptake or globally suppress GPX4 activity risk inducing significant nephrotoxicity. This physiological constraint suggests that the therapeutic window for conventional, systemically administered ferroptosis inducers may be markedly narrow. To achieve clinical viability, therapies must aim to exploit metabolic bottlenecks that are specific to RCC cells, rather than suppressing ferroptosis defense mechanisms that are highly conserved and essential in adjacent healthy tissues. Thus, off-target ferroptosis in normal renal tubular compartments should be considered a major dose-limiting safety concern when developing systemic ferroptosis-inducing therapies for RCC.

To mitigate these renal safety concerns and improve the therapeutic index, recent preclinical investigations have explored targeted delivery systems. Tumor microenvironment (TME)-responsive nanomedicines offer a potential strategy to spatially restrict ferroptosis induction. For example, nanoparticles engineered to release ferroptosis-inducing payloads exclusively under the specific physiological conditions of clear cell RCC (ccRCC) may minimize drug exposure to normal tubular cells (121). Additionally, lipid-based nanoparticles and advanced nanomedicine platforms are currently being evaluated for their capacity to enhance targeted drug accumulation within renal tumors while sparing functional nephrons (122, 123). By coupling ferroptosis-inducing agents with sophisticated delivery vehicles, it may be possible to induce targeted membrane damage in RCC cells without compromising overall renal function. These localized delivery approaches likely represent a necessary direction for the safe clinical translation of ferroptosis-based oncology treatments.

Beyond delivery systems, achieving a clinically acceptable therapeutic index will require multi-pronged strategies that exploit the unique metabolic vulnerabilities of RCC subtypes, particularly chromophobe RCC (chRCC). First, tumor-selective ferroptosis can be enhanced by targeting pathways that are preferentially essential in cancer cells but dispensable in normal kidney. For example, the ferroptosis suppressor protein 1 (FSP1) has emerged as a subtype-specific dependency in chRCC; pharmacological inhibition of FSP1 induces ferroptosis in chRCC models while potentially sparing normal renal tubular cells (68). Second, the development of tumor microenvironment-responsive nanocarriers can spatially restrict ferroptosis induction to the tumor bed, as demonstrated in ccRCC models (121, 123). Third, biomarker-guided patient selection—using redox phospholipidomics or lipid peroxidation signatures—will identify RCC patients whose tumors already operate near the ferroptotic threshold, thereby improving the risk-benefit profile. Additionally, newer ferroptosis-inducing agents with improved drug-like properties, such as GPX4 degraders or FSP1 inhibitors, are under active development and may offer a wider therapeutic window than first-generation compounds (105). Collectively, these strategies suggest that the therapeutic window for ferroptosis-based RCC therapy, while narrow for systemically administered conventional agents, can be meaningfully widened through rational combination of tumor-targeted delivery, subtype-specific vulnerabilities, and precision biomarker stratification.

### 5.3 Biomarkers, intratumoral heterogeneity, and future trial designs

Despite the mechanistic rationale for ferroptosis induction in RCC, clinical translation is constrained by substantial inter- and intra-tumoral heterogeneity (124). Within a single tumor mass, spatial variations in oxygen tension, nutrient availability, and immune infiltration establish distinct metabolic microenvironments (125, 126). In ccRCC, for example, immunometabolic coevolution has been shown to create localized niches characterized by unique redox and lipid states (127). Consequently, ferroptosis susceptibility is rarely uniform across an entire tumor. While therapeutic induction may effectively eradicate highly vulnerable subclonal populations, it might spare adjacent cells residing in microenvironments with robust ferroptosis suppressor capacities. Together, these observations suggest that spatial heterogeneity supports the notion that achieving complete tumor eradication through ferroptosis monotherapy may prove difficult in clinical settings.

In parallel, the efficacy of ferroptosis-inducing agents is likely limited by cellular metabolic plasticity. Cancer cells possess the capacity to dynamically reprogram their lipid metabolism and redox pathways in response to therapeutic stress (128, 129). Experimental models demonstrate that tumor cells can evade ferroptosis by upregulating alternative antioxidant systems or by remodeling their phospholipid composition to deplete peroxidizable substrates (130, 131). Such adaptive rewiring suggests that acquired resistance to ferroptosis induction may develop rapidly. Accordingly, sustained clinical responses will likely require combinatorial strategies designed to concurrently target the primary ferroptosis pathway and anticipated compensatory metabolic circuits.

Beyond heterogeneity and adaptive resistance, patient stratification represents another major translational hurdle. Ferroptosis susceptibility is an emergent, network-level phenotype. Static measurements of individual genes or proteins—such as GPX4 or iron transporter expression—generally fail to accurately reflect the dynamic peroxidation potential of a tumor cell. Instead, robust prediction of therapeutic response will likely depend on the development of composite biomarker signatures, which integrate data across lipidomic and metabolic platforms (132). Recent advances in redox phospholipidomics offer a more direct assessment of ferroptotic signaling by quantifying specific oxidized lipid species *in vitro* and *in vivo (*133). Incorporating these sophisticated analytical approaches into early-phase clinical trials may facilitate the identification of patient cohorts most likely to benefit from ferroptosis-targeted interventions.

A related source of uncertainty concerns the interpretation of preclinical ferroptosis research is frequently complicated by experimental model constraints. Standard *in vitro* cell culture media generally do not replicate the physiological nutrient, iron, and lipid availabilities found in human tumors. These artificial culture conditions can significantly alter cellular lipid composition and distort baseline ferroptosis sensitivity (134, 135). Consistent with this concern, studies have shown that physiological culture media can reduce metabolic artifacts and alter lipid peroxidation-related phenotypes in cancer models (136). This limitation is particularly critical for understudied subtypes like chRCC, where genetically faithful and metabolically accurate models remain scarce. For this reason, future research efforts should prioritize the use of physiologically relevant media and complex three-dimensional or *in vivo* models. By aligning metabolically accurate experimental design with the longitudinal tracking of lipidomic biomarkers, future clinical trials can more rigorously evaluate the viability of ferroptosis induction as a precision medicine strategy in RCC.

Extending this issue to the broader evidence base, current findings remain uneven across experimental systems, which may partly explain why ferroptosis sensitivity is not always reproducible across model contexts. Many ferroptosis-related findings in RCC are derived from conventional two-dimensional cell culture models or computationally inferred multi-omics signatures, whereas subtype-specific *in vivo* validation remains limited. Differences in culture media composition, oxygen tension, lipid availability, iron handling, and immune contexture may substantially alter ferroptosis sensitivity. Recent work further suggests that lipidome remodeling in three-dimensional growth conditions and xenograft tumors can reduce sensitivity to GPX4 inhibition, underscoring the need to validate ferroptosis phenotypes beyond standard monolayer culture (125). Therefore, findings obtained in one RCC subtype or model system should not be directly extrapolated to all RCC contexts. Future studies should compare ferroptosis responses across metabolically faithful cell models, patient-derived organoids, and *in vivo* platforms before ferroptosis induction is advanced as a broadly applicable therapeutic strategy (137).

Taken together, these experimental and translational limitations indicate that the clinical development of ferroptosis-inducing strategies in RCC remains at an early stage. Although ferroptosis-related therapeutic concepts have begun to enter early-phase oncology trials, canonical ferroptosis-inducing regimens have not yet established a defined clinical role in RCC (107, 138). In this context, future phase I studies in RCC should prioritize safety, pharmacodynamic validation, and biomarker-guided patient selection rather than immediate efficacy claims. A rational early-phase framework would include dose-escalation cohorts in advanced RCC patients with careful monitoring of renal function, AKI-related signals, and systemic oxidative toxicity. Correlative analyses should incorporate lipid peroxidation markers, glutathione pathway activity, iron-handling signatures, and ferroptosis suppressor pathways such as GPX4 and FSP1. Such a design would help determine whether ferroptosis modulation can be achieved within an acceptable therapeutic window before expansion into subtype-specific cohorts, including chRCC.

To clarify the translational relevance of these mechanisms, **Table 3** summarizes major ferroptosis-related therapeutic entry points together with their clinical development considerations. Representative agents and tool compounds include system Xc− inhibitors such as erastin and sulfasalazine, GPX4-directed agents such as RSL3 and FIN56-like compounds, iron-modulating approaches, and emerging FSP1-targeted strategies. At present, these approaches should be interpreted mainly as preclinical or early translational concepts in RCC rather than established clinical treatments.

**Table 3:** Ferroptosis-related therapeutic targets and biomarker-guided translational considerations in renal cell carcinoma.

Targeted pathway	Therapeutic strategy concept	Mechanistic rationale	Key translational considerations
Cysteine and glutathione metabolism (59)	Restriction of cystine uptake or glutathione synthesis	Impairs lipid peroxide detoxification and increases oxidative vulnerability	Tumor specific cysteine dependence, alternative cysteine supply routes
Lipid peroxide detoxification (109)	Disruption of phospholipid hydroperoxide reduction	Accelerates membrane damage under oxidative stress	Selectivity and renal tissue safety
Iron handling (114)	Expansion of labile iron pool or disruption of iron storage	Increases catalytic pressure for lipid peroxidation	Systemic iron regulation and toxicity
Mitochondrial stress (90)	Enhancement of oxidative pressure in metabolically stressed tumors	Amplifies ferroptosis susceptibility	Context dependent efficacy
Lipid remodeling (109)	Enrichment of oxidizable membrane phospholipids	Expands substrate pool for lipid peroxidation	Metabolic plasticity and adaptation

Building on the therapeutic targets summarized in **Table 3**, **Figure 4** illustrates how ferroptosis-inducing strategies may be aligned with RCC metabolic dependencies, biomarker selection, and renal safety monitoring, rather than applied as a uniform treatment strategy for all RCC patients (105).

## 6 Conclusion

Renal cell carcinoma is now being identified as a metabolically defined class of malignancies where the tumor’s survival and growth is strongly linked to how the tumor adapts to maintain redox homeostasis, mitochondrial function, and membrane stability during prolonged periods of stress. All forms of renal cancer share common features including metabolic reprogramming that allow them to grow in areas with variable amounts of oxygen, variable sources of nutrients and increased oxidative pressures. These adaptations enable the tumor to survive but they also limit the tumor’s ability to grow and create new blood vessels (angiogenesis) or evade the host’s immune response. Ferroptosis, a regulated form of programmed cell death, directly targets some of the limitations of tumor cells by transforming abnormal iron metabolism, abnormal lipid synthesis, and decreased levels of antioxidants into permanent membrane damage.

This review provides a mechanism-based understanding of ferroptosis as a newly emerging phenotypic expression of programmed cell death resulting from the integration of multiple factors including available iron, lipid membrane composition, oxidative stress produced by mitochondria, and the capability to detoxify lipid peroxides. Disrupting this balance of factors may cause ferroptotic collapse even in tumors that are resistant to programmed cell death (apoptosis), and have developed resistance to most traditional chemotherapeutic agents. An important aspect of ferroptosis is that it primarily involves cellular mechanisms that are independent of the pathways involved in promoting angiogenesis and activating immunosuppressive pathways, suggesting that ferroptosis is a complementary therapeutic strategy to other treatment options and not a replacement for existing treatment options.

Chromophobe renal cell carcinoma is one example of a renal cancer type that has characteristics that make it an attractive candidate for targeting ferroptosis based treatments. Chromophobe renal cancers often contain abnormal mitochondria, have reduced oxidative metabolism, elevated oxidative pressure, and compensate for these defects by relying heavily on antioxidants to buffer against oxidative damage. Given the high degree of dependence on antioxidants, these tumors likely exist close to the maximum amount of lipid peroxides that can be controlled. Therefore, selective disruption of ferroptosis suppressor pathways in chromophobe renal cell carcinomas could provide additional avenues to target this tumor type that are not currently available through the use of systemic therapies.

While there is no definitive evidence supporting the utility of ferroptosis oriented treatments for chromophobe renal cell carcinoma, the biological basis of this approach is strong enough to support further study in experimentally tractable models and the design of carefully crafted clinical trials.

However, several challenges associated with developing effective clinical applications of ferroptosis need to be acknowledged. Ferroptosis is a complex process that results from the interplay of many different molecular networks and the complexity of renal cancer biology and its metabolic plasticity will likely pose significant challenges to identifying the correct patients for treatment. Developing reliable biomarkers that accurately capture changes in the redox state and lipid composition of the tumor will be critical to determining whether patients will benefit from ferroptosis inducing therapies. Moreover, it will be essential to develop therapeutic strategies that take into account anticipated compensatory adaptations of the tumor and to minimize the potential toxicities of ferroptosis inducing therapies. Finally, safety issues must be taken seriously because of the high degree of reliance of normal kidney tissues on their intact redox and lipid repair mechanisms. Therefore, developing strategies to selectively target the tumor’s metabolic bottlenecks will be required to ensure that the majority of the toxicities of ferroptosis inducing therapies are confined to the tumor. Importantly, as discussed in the preceding section, such selectivity can be achieved through tumor-responsive nanocarriers, subtype-specific pathway inhibition, and biomarker-guided patient enrichment, thereby moving beyond a one-size-fits-all approach to ferroptosis induction. Furthermore, ferroptosis inducing therapies should be combined with other therapeutic strategies that have been shown to be effective in treating renal cell carcinoma in order to minimize systemic toxicities.
